# Disappearance of Acquired Hemophilia A after Complete Remission in a Multiple Myeloma Patient

**DOI:** 10.4274/tjh.2016.0146

**Published:** 2017-06-01

**Authors:** Vanessa Innao, Alessandro Allegra, Rosalba Morreale, Sabina Russo, Caterina Musolino

**Affiliations:** 1 Messina University Faculty of Medicine, Department of Human Pathology and the Adult Developmental, Division of Hematology, Messina, Italy

**Keywords:** Acquired hemophilia, Multiple myeloma, Factor VIII, Autoantibody, Coagulation disorder

## To The Editor,

Acquired inhibitors of blood coagulation are endogenously produced pathologic substances that either react directly with clotting factors or inhibit their reactions. Acquired hemophilia A (AHA) is caused by polyclonal inhibitory immunoglobulins G (predominantly IgG1 and IgG4) against factor VIII (FVIII). They react with A2, A3, or C2 domains of the FVIII molecule, blocking its interactions with active factor IX, phospholipids, and von Willebrand factor. A disturbed proportion of CD4^+^ Th1 to Th2 cells plays a role in autoantibody production and reactivity [1,2]. FVIII inhibitors have most often been associated with autoimmune diseases, drugs, immunosuppressive therapies, malignant neoplasms, or obstetric accidents in the postpartum period [3].

Here we report a rare case of a patient with AHA and multiple myeloma, where the disappearance of the hematologic malignancy induced by chemotherapy resulted in a regression of the coagulation disorder.

A 67-year-old man came under our observation for plasmacytoma. Significantly, his past medical history included both high blood pressure and hip replacement. His concomitant medication was only lacidipine. In 2009 the patient underwent cholecystectomy without bleeding. In 2014, he underwent a surgical excision of basal cell carcinoma in the pectoral region without bleeding. 

In 2014 the patient was diagnosed, at a different hematological center, with congenital mild hemophilia A and monoclonal gammopathy of undetermined significance IgG kappa. At that time, FVIII was 29.1%. He also underwent a desmopressin test, which displayed an increase of FVIII activity (15% at basal level) up to 90% after 60 min. Elevation of IgG (2319 mg/dL) was present, while the rest of the immunoglobulins were within normal values. IgG k-type monoclonal protein was detected in the serum and urine.

In 2015, a diagnosis of plasmacytoma resulted from a biopsy of abnormal tissue performed during vertebroplasty for a pathological L3 fracture. The orthopedic intervention was performed with premedication with recombinant FVIII. He came, therefore, to our attention presenting with 28% FVIII activity, prothrombin time within normal limits, and prolonged activated partial thromboplastin time (44.1 s).

Serum protein electrophoretic analysis showed a monoclonal peak within γ-globulin, increased IgG, and suppression of all other components. Serum protein electrophoresis showed a monoclonal free k-light chain.

Bone marrow biopsy, performed after infusion therapy of recombinant FVIII, showed plasma cells at 50%. Therefore, a diagnosis of multiple myeloma IgG kappa, stage IIA Durie-Salmon, I ISS, was made. He began treatment with intravenous bortezomib and oral melphalan and prednisone, associated with monthly infusions of bisphosphonates, for 6 cycles in total. Revaluation of the disease documented stringent complete response (sCR), but we also witnessed the normalization of blood coagulation parameters, while FVIII activity was 98%. The patient was then mobilized with high-dose cyclophosphamide and autotransplanted, retaining the sCR to this day.

Diagnosis of AHA can be difficult. It is often unrecognized or misdiagnosed as other acquired hemorrhagic disorders, such as disseminated intravascular coagulation, acquired inhibitors against von Willebrand factor, and acquired factor XIII deficiency [4].

The association of acquired hemophilia and multiple myeloma is extremely rare; there are only four such reports in the literature [5,6,7,8]. However, prompt diagnosis of this acquired bleeding disorder is essential for management, aimed at hemorrhage control and inhibitor suppression. In our case, the misdiagnosis led not only to a delayed diagnosis of the real coagulation disorder, but probably also to a delay even of hematologic malignancy, effectively responsible for the coagulation dysfunction.

As said above, both solid tumors and hematologic malignancies, especially lymphoproliferative diseases, are able to predispose or be associated with this antibody’s growth [9].

A small number of works investigated the genetic basis of AHA, due to immune tolerance breakdown. Pavlova et al. [10] pointed out higher frequencies of human leukocyte antigen class II alleles DRB*16 (odds ratio [OR]: 10.2) and DQB*0502 (OR: 2.5). In a different work, the CTLA-4 +49G allele was increased in these patients with an OR of 2.17 and these data were not confirmed in congenital hemophilia.

In conclusion, it is clear that in our case the reestablishment of immunocompetence, complete remission of multiple myeloma, and disappearance of AHA are closely related events. In fact, previous works have demonstrated that the treatment of the underlying cancer led to the disappearance of the inhibitor in 22% of patients [11]. Finally, as multiple myeloma is a treatable but not curable disease, it will be interesting to keep closely monitoring coagulation in the patient in the case of relapse, to evaluate if it can lead to the reappearance of FVIII inhibitors.
